# Antibacterial effect on microscale rough surface formed by fine particle bombarding

**DOI:** 10.1186/s13568-022-01351-8

**Published:** 2022-01-31

**Authors:** Tomoko Nishitani, Kyosuke Masuda, Soma Mimura, Takahiko Hirokawa, Hitoshi Ishiguro, Masao Kumagai, Takeshi Ito

**Affiliations:** 1 Surf Technology Co., Ltd, 4-1-83 Onodai, Minami-ku, Sagamihara, Kanagawa 252-0331 Japan; 2 Graduate School of Science and Engineering, Kansai University, 3-3-35, Yamate-cho, Suita, Osaka 564-8680 Japan; 3 Kanagawa Institute of Industrial Science and Technology, Shimoimaizumi, Ebina, Kanagawa 705-1243-0435 Japan

**Keywords:** Fine particle bombarding, Antibacterial effect, Surface shape, Microscale roughness

## Abstract

**Abstract:**

Fine particle bombarding (FPB) is typically utilized to modify metal surfaces by bombarding them with fine particles at high-speed. The diameters of the particles range from several to tens of micrometers. FPB forms fine microscale concavities and convexities on a surface. As FPB-treated surfaces are widely used in the food industry, the influence of bacteria on their surface must be considered. In this study, we examined the antibacterial activity of microscale rough surfaces formed by FPB. We applied FPB to a stainless-steel surface and evaluated the antibacterial effect of FPB-treated surfaces based on JIS Z 2801 (a modified test method from ISO 22196:2007). Our results indicated that the FPB-treated surfaces (FPB-1 (avg. pitch: 0.72 µm) and FPB-2 (avg. pitch: 3.56 µm)) exhibited antibacterial activity both against *Escherichia coli* and *Staphylococcus aureus*.

**Graphical Abstract:**

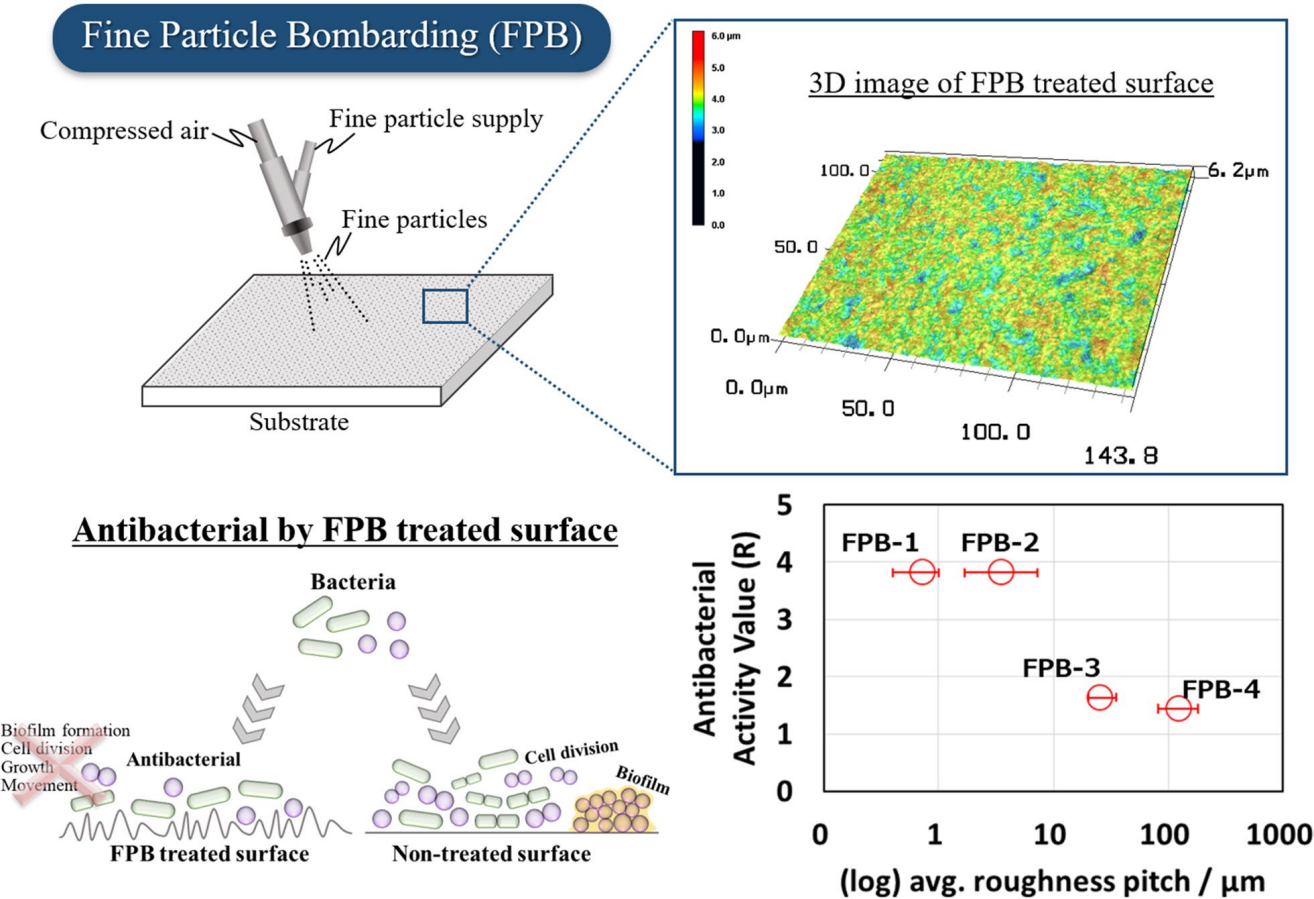

**Supplementary Information:**

The online version contains supplementary material available at 10.1186/s13568-022-01351-8.

## Introduction

Numerous antibacterial agents have been developed to combat infections of bacterial origin. Conventional antibacterial agents are generally classified as organic or inorganic compounds. Organic antibacterial agents include natural compounds such as catechin (Silva et al. 2016) and drugs such as penicillin, which immediately and pharmacologically act against bacteria (Watanabe et al. 2013; López-Gigosos et al. 2017). Inorganic antibacterial agents include compounds such as silver, zinc, and copper, and photocatalysts such as titanium dioxide (Thurman et al. 1989; Saidin et al. 2021). Although the antibacterial activity of these compounds has been attributed to active metal ions and reactive oxygen species, the detailed mechanism remains unclear.

In particular, organic antibiotics pose a global risk for human health because they give rise to drug-resistant bacteria (Townsend et al. 1985; Sasatsu et al. 1990). The number of deaths due to drug-resistant bacteria has increased worldwide. The annual death toll caused by drug-resistant bacteria is 35,000 in the United States and more than 33,000 in Europe (Redfield 2019). It is estimated that the number of global deaths caused by drug-resistant bacteria will reach 10 million by 2050 (O’Neill 2016). This number is expected to exceed the deaths due to cancer.

Recently, the materials that exhibit antibacterial effects due to their structural properties have attracted considerable attention. The possible effects of such materials on drug-resistant bacteria have been investigated. Ivanova et al. reported that the nano-sized structures on the wings of cicadas and dragonflies exhibit antibacterial activity (Ivanova et al. 2012, 2013; Pogodin et al. 2013). Following this report, various artificial nanostructures with antibacterial and sterilization properties have been actively researched (Zouaghi et al. 2019; Hasan et al. 2020; Mann et al. 2014). Micro-sized patterns also show antibacterial properties. Brennan et al. developed an antibacterial film (Sharklet™) using a silicone elastomer and reported the antibacterial effects of the film against various bacteria. The film imitated scales on shark skin with micro-order irregularities (Chung et al. 2007; Reddy et al. 2011). The technology of imitating the biologically advantageous structures of organisms in nature that have evolved over time is referred to as biomimetics. A major advantage of using biomimetics-based antibacterial materials is that the need for antibacterial drugs could be reduced. The antibacterial effects of biomimetic materials are semipermanent and last as long as the shape of the material is maintained. Jindai et al. confirmed the antibacterial property of an artificially built nanopillar structure (Jindai et al. 2020). In this case, after bacteria attached to the artificial nanopillar, the bacterial cell membrane was damaged owing to the interaction between the bacteria and pillar. Then, intracellular fluid leaked out, leading to bacterial death. However, the details of the bactericidal mechanism of nanostructures are still unclear. Yamashita et al. suggested that antibacterial and antifungal properties are observed at the microscale (Yamashita et al. 2017). These reports focus on the antibacterial effects of regular surface topography at the nanometer to micrometer scale. In addition, it is likely that each of the structures described above has a different underlying antibacterial mechanism.

Thus far, artificial nanostructures have been produced using a microelectromechanical system (MEMS) process such as wet etching or dry etching coupling with lithography techniques (Carman et al. 2006). However, etching has a significant environmental impact owing to the chemicals used in the process and the amount of labor required for their disposal. Dry etching requires expensive devices and ancillary equipment, such as gas removal devices, which increase cost and limit the size of substrates. In our previous studies, we used fine particle bombarding (FPB) (Tamura et al. 2021; Morita et al. 2020; Ohue et al. 2008; Morita et al. 2013) to achieve microscale roughness. In FPB, a microscale rough surface is formed by bombarding fine particles, such as ceramics, onto a metal surface. Here, the particles are accelerated using a compressible gas. Deforming a surface of the base material can improve the wear resistance and sliding properties of machine parts. Furthermore, it improves the endurance limit by applying compressive residual stress to surface layers. One advantage of FPB is that it can treat a large surface area of up to tens of square meters. Additionally, the fine particle materials can be reused, thereby limiting the environmental impact and finally, the processing method or device settings do not need to be changed for varying shapes of the processed product. This makes it possible to handle complicated three-dimensional shapes. Given the short processing time, ease of handling, and low cost of FPB, microscale asperities can be easily obtained. One benefit of this technique is a reduction in both the adhesion and friction of powders such as flour on the modified surface. When a powder is present on an FPB-treated surface, the convex parts of fine asperities and the powder are in point contact. This reduces adhesion and friction compared to mirror-polished stainless-steel surfaces. Consequently, FPB is widely used as a surface modification treatment for equipment in the food industry. To the best of our knowledge, there are no reports that consider the dependence of the antibacterial effect on the surface roughness of FPB-treated stainless steel. Thus, potential issues regarding the behavior of bacteria on these surfaces must be investigated. For example, the increased surface area due to microscale roughness may present an advantage to the growth of bacteria.

In this study, we prepared FPB-treated stainless steel and evaluated their wettability and antibacterial activities. Compared to untreated stainless steel, FPB-treated stainless steel shows strong antibacterial effects both against *Escherichia coli* (*E. coli*) and *Staphylococcus aureus* (*S. aureus*), demonstrating at least a three-fold decrease in the viable cell count. The FPB-treated stainless-steel surface consists of fine microscale concavities and convexities. However, unlike organic antibacterial agents, an antibacterial surface generated by FPB does not have immediate effects. If the surface profile is maintained, the antibacterial effect remains intact. Furthermore, as described above, the treatment is simple and can be performed over a large area. This makes it suitable for use in the food and medical industries.

## Experimental methods

### Chemicals and materials

Propidium iodide (PI), sodium chloride, potassium chloride, ethylenediamine-N, N, N’,N’-tetraacetic acid dipotassium salt dihydrate (EDTA-2K), and dimethyl sulfoxide (DMSO) were purchased from Fujifilm Wako Chemicals (Tokyo, Japan). SYTO 9 stain and Bacto Tryptone were obtained from Thermo Fisher Scientific (Tokyo, Japan). Distilled water was used in this study.

Four types of test pieces were prepared using a SUS304 #400 polished substrate (50 mm × 50 mm, thickness: 1.0 mm) as a base material which was then subjected to FPB. A polished SUS304 #400 was also used as a control. The test pieces were named FPB-1, FPB-2, FPB-3, and FPB-4. The substrate was purchased from ZIP MOTOR PRO (Osaka, Japan). The following fine particle materials were used for FPB: tungsten carbide (WC); median size = 0.7–1.2 µm for FPB-1 (Kojundo Chemical Lab. Co., Ltd., Saitama, Japan), Densic® (silicon carbide: SiC); median size = 3.7–4.5 µm for FPB-2 (Showa Denko K.K., Tokyo, Japan), FHB series (FHB); median size = 38–53 µm for FPB-3 (Potters-Ballotini Co., Ltd. Ibaraki, Japan), and steel shots (Steel); median size = 425–710 µm for FPB-4 (Ikk Shot Co., Ltd., Aichi, Japan).

### Formation of microdimples by FPB

Stainless-steel surfaces with various roughness values were formed using FPB (Additional file 1: Figure S1). The surface roughness depended on the composition and size of the fine particle material. Additional file 5: Table S1 summarizes the conditions of each FPB process. The substrate was treated using a blast machine (Pneuma Blaster FDQ-2S-L101, Fuji Manufacturing Co., Ltd., Japan). The fine particle materials were mixed with a compressible gas and bombarded onto the substrate surface at a high speed (150–200 m/s). This led to plastic deformation and the formation of irregular and fine asperities referred to as microdimples on the treated surface. A laser microscope (VK-X100, KEYENCE, Japan) was used to evaluate the roughness of the FPB-treated surfaces.

### Measurement of contact angle on FPB-treated surfaces

FPB can control the wettability of a treated surface. A few reports (Pan et al. 2019; Nakade et al. 2018; Qian et al. 2019) indicated that the wettability of a surface with a nanopillar structure was related to the viable cell rate. Thus, we evaluated the wettability of the FPB-treated test pieces by measuring their water contact angles (WCAs) using a contact angle meter (DMo-701, Kyowa Interface Science, Japan) with 1.5 µL of purified water droplets. Thereafter, an antibacterial property test was conducted using the test pieces.

### Antibacterial property test

*E. coli* (NBRC3972) and *S. aureus* (NBRC12732) were used for the antibacterial property test. The test was conducted using the FPB-treated test pieces and the control as a reference (number of samples (N) = 3 for tests). The test was performed using a protocol based on the Japanese Industrial Standards (JIS) Z 2801 method, which is a modified test method taken from ISO 22196:2007. JIS Z 2801 defines an antibacterial effect as having an antibacterial activity score of 2.0 or more after a 24 h inoculation.

The concentration of *E. coli* (NBRC3972) was 3.5 × 10^5^ CFU/mL (CFU; colony forming unit). The area of all the test pieces was 1.6 × 10^3^ mm^2^. A test solution (0.4 mL) containing viable bacteria was introduced to each test piece. Next, each test piece was covered with a sterilized film (Esclinica Pack L, Sekisui Chemical Co. Ltd., Japan). Then, the temperature of the test pieces was maintained at 35 ℃ for 8 h for cultivation. The test solution was rinsed away using a sterilized saline solution (9.6 mL). A volume of 10 mL was analyzed to count the viable bacteria, as described below. The concentration of viable bacteria in the test solution was obtained to estimate the antibacterial property using a counting sheet (JNC, Japan). *S. aureus* (NBRC12732) was grown using the same method described in JIS Z 2801.

The concentration of *S. aureus* was 2.7 × 10^6^ CFU/mL for 6 and 12 h experiments and 2.5 × 10^6^ CFU/mL for 0 and 24 h experiments. A test solution (0.1 mL) containing viable bacteria was coated on each test piece. Then, the test pieces were covered with a sterilized polypropylene film (KOKUYO, Japan). The temperature of the test pieces was maintained at 35 ℃ for 0, 6, 12, and 24 h for cultivation. The viable cell count after culturing was measured using the colony formation method described in JIS Z 2801. The antibacterial activity (R) was calculated using Eq. 1.

1 $${\text{Antibacterial activity }}\left( {\text{R}} \right) \, = {\text{ log }}\left( {\text{Number of viable bacteria after culture on untreated test piece}} \right) - {\text{ log }}\left( {{\text{Number of viable bacteria after culture on FPB}} - {\text{treated test piece}}} \right)$$

### Live/dead assay method

*E. coli* (RP437) cells were grown in tryptone broth (1% Bacto Tryptone, 0.5% NaCl) at 30 °C with shaking at 170 rpm until OD_600_ = 0.6, where OD_600_ denotes the turbidity of *E. coli* at 600 nm. Cultured cells were suspended in a motility buffer twice (10 mM potassium phosphate buffer with pH = 7.0; 0.1 mM EDTA-2 K with pH = 7.0; 10 mM NaCl; 75 mM KCl). The cell suspension was diluted with the motility buffer to OD_600_ = 0.2.

Cells were stained with SYTO 9 and PI to perform a cell membrane damage test. A diluted cell suspension (1 mL) was mixed with a SYTO 9–DMSO solution (3.3 mM) and a PI–DMSO solution (10 mM). After mixing, the cell suspension was allowed to stand for 15 min.

After the cell suspension (20 µL) was dropped on to the test pieces, a coverslip (18 mm × 18 mm, thickness: 0.15 mm) was placed over them and fixed using double-sided tape (0.1 mm thick). The dropped suspension was pushed out by the coverslip to ensure that the cell suspension covered the entire area of the FPB-treated surfaces. The chamber was held for 1 min to allow cells to adhere to the test piece surfaces.

The membrane damage in the *E.coli* cells that adhered to the FPB-treated surfaces was assessed by performing fluorescence microscopy (Eclipse Ni-U, Nikon, Tokyo, Japan) using a 40 × objective lens (CFI S Plan Fluor ELWD 40XC, N.A.0.6, Nikon, Japan) and a 2.5 × C-mount relay lens (VM2.5X, Nikon, Japan). *E. coli* cells stained with SYTO 9/PI were illuminated with epifluorescence from a mercury lamp. We used the GFP HQ filter set (excitation filter: 470/40 nm, dichroic mirror: 495 nm, emission filter: 525/50 nm; Nikon, Tokyo, Japan) to observe SYTO 9 fluorescence and the mCherry filter set (excitation filter: 570/40 nm, dichroic mirror: 600 nm, emission filter: 645/75; Nikon, Tokyo, Japan) to observe PI fluorescence. Fluorescence images were captured from 5 to 60 min using a charge-coupled device camera (DMK33UX265; The Imaging Source, Germany) (number of images (N) = 3). Excitation light was irradiated for 1 s for each capturing process to reduce photobleaching.

The time course of the active cell ratio, which was calculated as the number of SYTO-9-stained cells divided by the number of attached cells (green-stained and red-stained cells), was analyzed using ImageJ (Schneider et al. 2012; Abramoff et al. 2004). Scanning Electron Microscopy (SEM: JSM 7500F, JEOL, Tokyo, Japan) was used to confirm the shape of bacteria attached to the FPB-treated surface. No additional treatment was considered for the observation apart from the drying of the sample after washing it with distilled water following the live/dead assay.

## Results

### Physical and physicochemical properties of FPB-treated surface

**Fig. 1:**
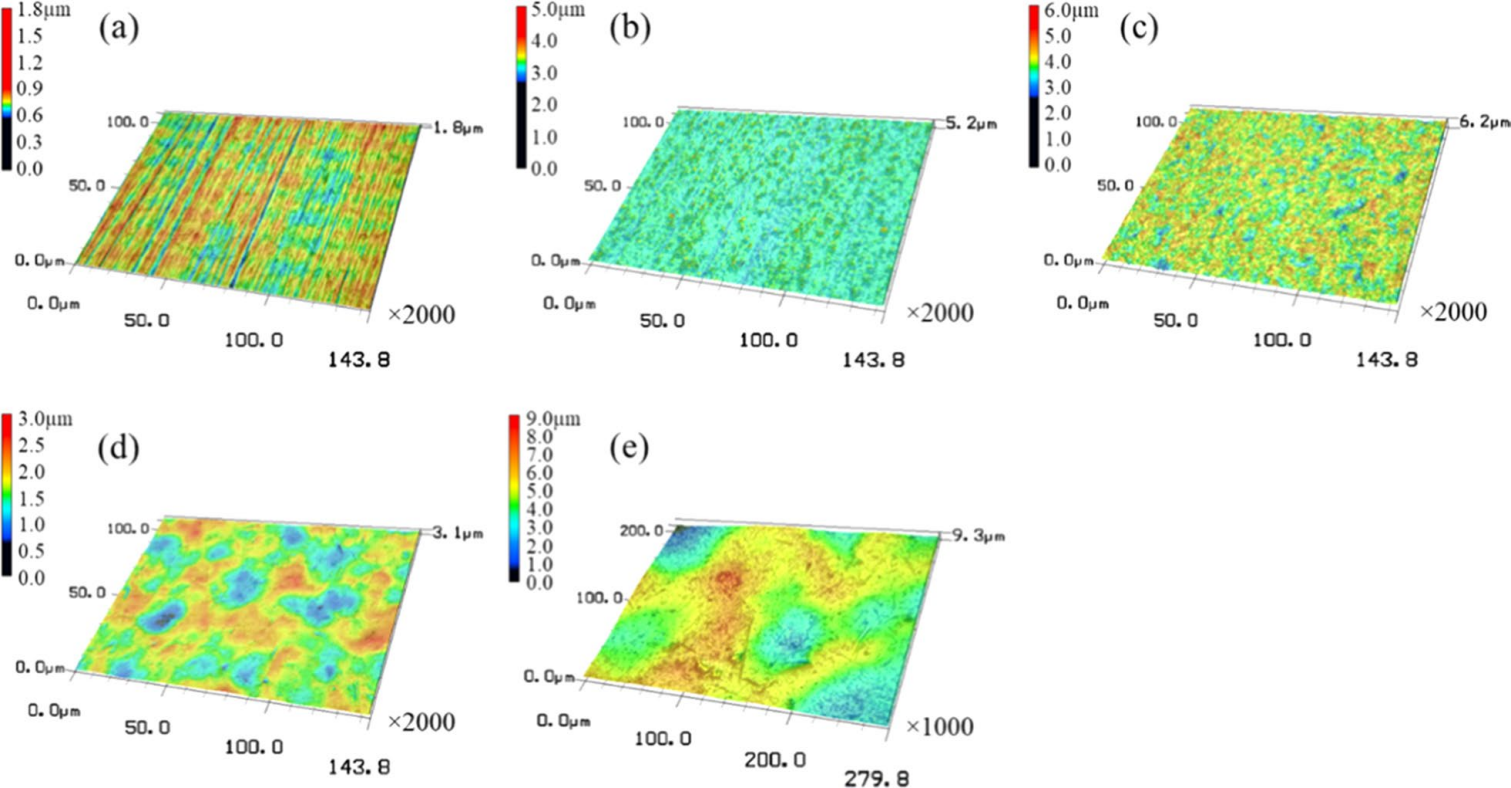
3D images of control and FPB surfaces. **a** Control, **b** FPB-1, **c** FPB-2, **d** FPB-3, and **e** FPB-4

**Fig. 2:**
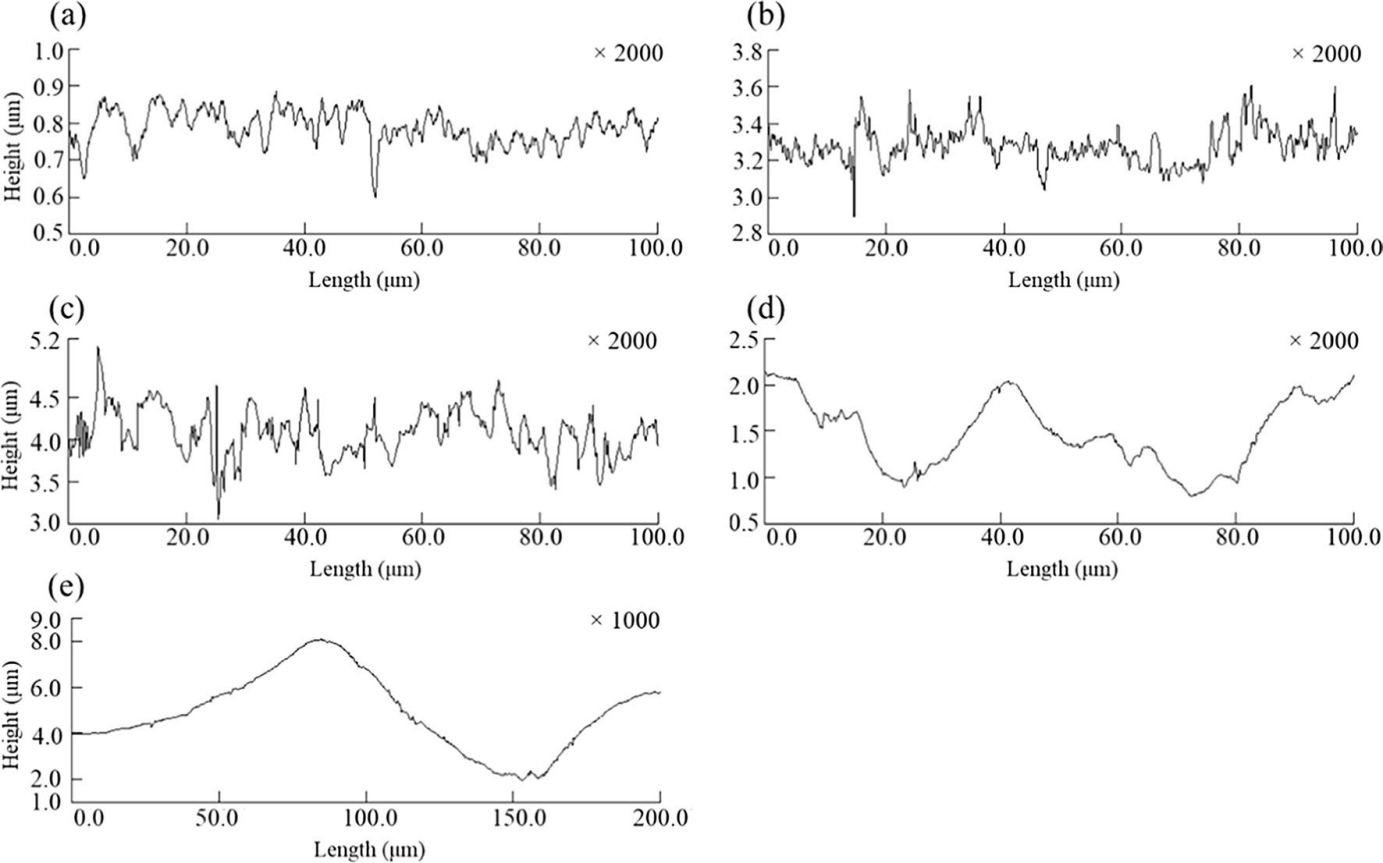
Cross-sectional profile curves of control and FPB-treated surfaces. **a** Control, **b** FPB-1, **c** FPB-2, **d** FPB-3, and **e** FPB-4.

Figure 1 shows the 3D surface images of the control and the four different types of FPB-treated surfaces. The images were obtained using a laser microscope at 1000 × magnification for FPB-4 and 2000 × magnification for all other test pieces. The height scale was different for each test piece. The SUS304 #400 polished surface (a) had polishing marks. However, these marks were not observed on all of the FPB-treated surfaces, which were rough surfaces of varying magnitude. It should be noted that the rough surfaces consisted of numerous dimples. Figure 2 illustrates the cross-sectional shape of the four types of FPB-treated surfaces. As the roughness of the FPB-treated surfaces was mainly due to plastic deformation, dimple-shaped concavities of different sizes were formed on the surfaces. In addition, ridges (convex structures) were formed around the concavities.

Table 1 shows the pitch between the concavities and convexities (roughness pitch: Additional file 2: Figure S2) and the depth of the concavities. Roughness was distributed across the entire surface owing to the random occurrence of plastic deformation. The roughness pitch and roughness depth shown in Table 1 were obtained from the cross-sectional curves at three random locations on one sample. The size of the dimples formed by FPB increased with the size of the fine particle material.

**Table 1:** Roughness of FPB-treated surfaces

Test piece	Pitch (µm)	Depth (μm)
FPB-1	0.4–1.0, avg. = 0.72	0.04–0.17, avg. = 0.10
FPB-2	1.7–7.3, avg. = 3.56	0.2–1.0, avg. = 0.51
FPB-3	20–35, avg. = 25.7	0.6–0.9, avg. = 0.77
FPB-4	81–183, avg. = 124.4	1.5–4.6, avg. = 3.10

**Fig. 3:**
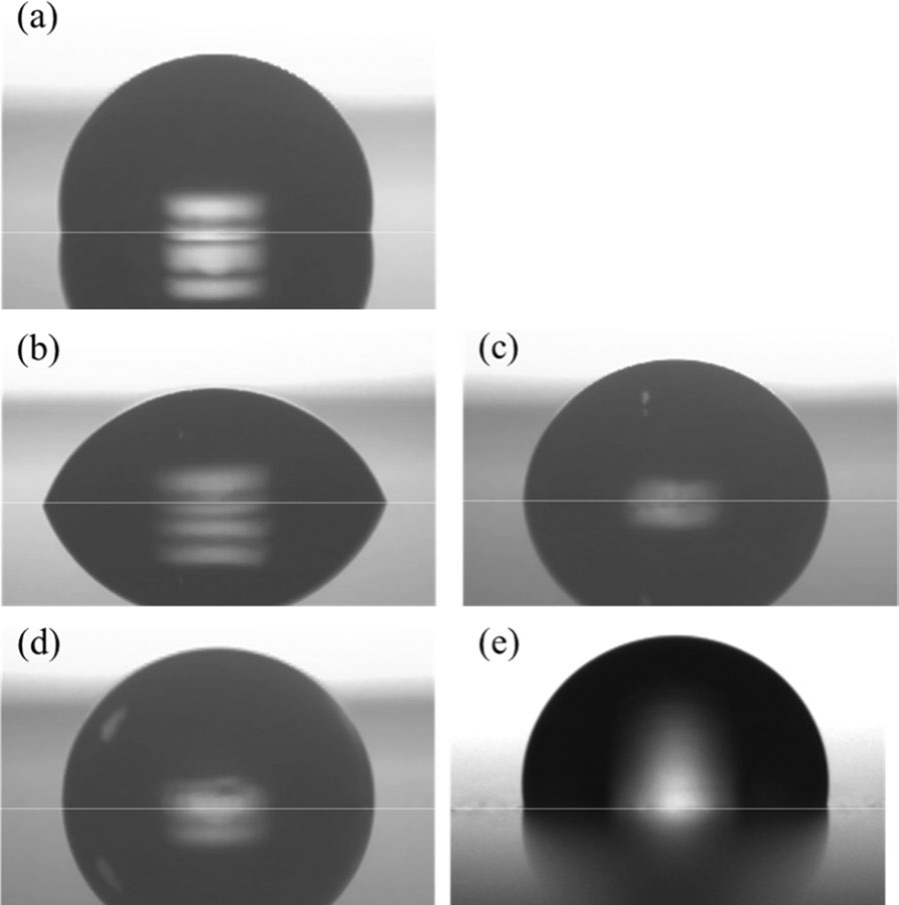
Images for evaluating the WCA of control and FPB-treated surfaces. **a** Control, WCA = 98.5°, **b** FPB-1, WCA = 65.5°, **c** FPB-2, WCA = 85.4°, **d** FPB-3, WCA = 90.0°, and **e** FPB-4, WCA = 92.3°

The images used for evaluating the WCA for each test piece are shown in Fig. 3. The WCA of the four FPB-treated surfaces ranged from 65.6° to 92.3°, which was lower than that of the control surface (98.5°). In this case, the smaller roughness pitch made lower WCA (Additional file 3: Figure S3).

The physical properties of a surface, such as the contact angle, have an effect on the surface free energy (Kitazaki and Hata 1971; Fowkes 1964; Peper et al. 1964; Tamai et al. 1967; Hamilton 1972). Usually, the surface free energy depends on the material specificity. However, it is possible to control the apparent surface free energy by altering the shape of a surface. When a liquid is dropped on to a solid surface, it experiences surface tension. A change in the contact angle caused by a change in the shape of the solid surface will have an effect on surface tension and consequently, surface free energy.

### Antibacterial property test against E. coli

Table 2 shows the results of the antibacterial property test against *E. coli*. The maximum viable cell count decreased rapidly in this experiment; therefore, the maximum test time was decreased to 8 h. The antibacterial activity scores were calculated from the viable cell count in the washout solution after the test. The scores for FPB-1 and FPB-2 were higher than or equal to those for FPB-3 and FPB-4, which indicates their antibacterial effects. The viable cell count concentrations for FPB-3 and FPB-4 were lower than those for the corresponding untreated test pieces. Although a certain degree of antibacterial effect was observed for FPB-3 and FPB-4, their antibacterial activity scores were lower than those for FPB-1 and FPB-2.

**Table 2:** Results of antibacterial property test for *E. coli*

Test piece	Bacterial count (CFU/mL)		R; antibacterial activity
Control	4.0 × 10^3^	avg. = 8.4 × 10^3^	–
	1.7 × 10^4^		
	4.2 × 10^3^		
FPB-1	< 1.0	avg. < 1.0	3.8
	< 1.0		
	< 1.0		
FPB-2	< 1.0	avg. < 1.0	3.8
	< 1.0		
	< 1.0		
FPB-3	3.7 × 10^1^	avg. = 2.2 × 10^2^	1.6
	4.0 × 10^2^		
	2.3 × 10^2^		
FPB-4	1.0 × 10^2^	avg. = 3.8 × 10^2^	1.4
	8.9 × 10^2^		
	1.5 × 10^2^		

Next, we examined the relationship between the roughness pitch and antibacterial performance of the four FPB-treated surfaces. The average roughness pitch for which an antibacterial effect was obtained was 0.72 µm for FPB-1 and 3.56 µm for FPB-2. The value of this pitch for FPB-3 and FPB-4 was uneven and 30 to several hundred times larger than that for FPB-1 and FPB-2. However, the results of the antibacterial property test for *E. coli* confirmed that FPB-3 and FPB-4 inhibited bacterial growth. These results showed that the antibacterial effect of the rough surface created via FPB was related to the surface roughness, and the antibacterial effect became stronger as the surface roughness decreased.

### Antibacterial property test against* S. aureus*

Table 3 shows the results of the antibacterial property test against *S. aureus*, using control as a reference, FPB-1 and FPB-2, which showed antibacterial effects for *E. coli*.

**Table 3:** Result of the antibacterial property test for *S. aureus*

Sample		Bacterial count (CFU/sample/cm^2^)	R; antibacterial activity
	Time (h)		
Control	0	1.3 × 10^4^	–
	6	1.1 × 10^4^	–
	12	2.9 × 10^3^	–
	24	6.2 × 10^3^	–
FPB-1	0	1.2 × 10^4^	–
	6	1.4 × 10^2^	1.9
	12	7.8 × 10^0^	2.3
	24	6.4 × 10^0^	3.0
FPB-2	0	1.3 × 10^4^	–
	6	2.4 × 10^2^	2.8
	12	9.5 × 10^–1^	3.2
	24	5.4 × 10^0^	3.5

The number of viable cells gradually decreased with time. In addition, FPB-1 and FPB-2 achieved an antibacterial activity score of 2.0 within 12 h, and FPB-2 showed a higher reduction rate of the viable cell count.

## Discussion

**Fig. 4:**
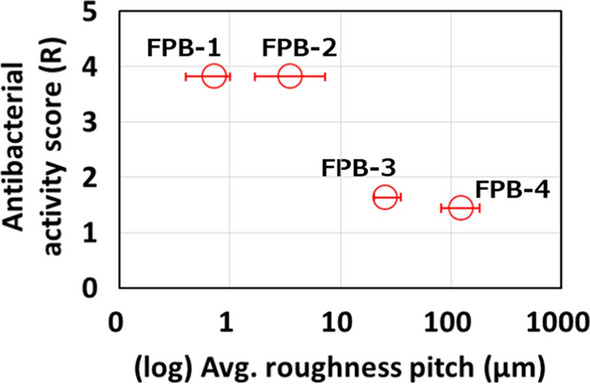
Relationship between the antibacterial activity score and roughness pitch of FPB-treated surfaces

**Fig. 5:**
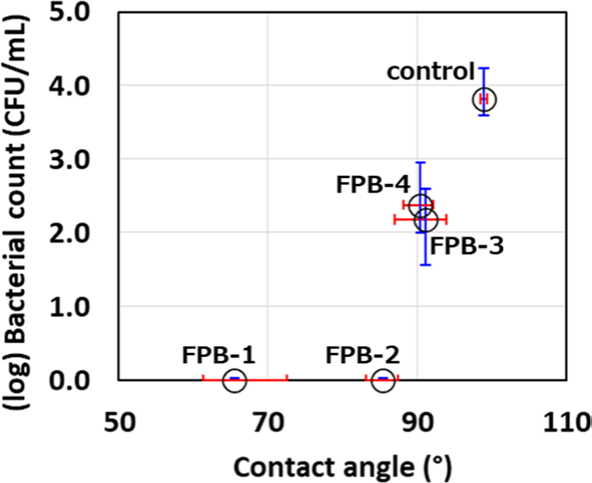
Relationship between the viable bacterial count and WCA on the control and FPB-treated surfaces

To discuss the antibacterial activity dependent with surface roughness, the relationship between the logarithm of the average roughness pitch of the dimples formed on each FPB-treated surface and antibacterial activity is shown in Fig. 4. According to the figure, a treated surface with dimples smaller than certain roughness pitch showed a strong antibacterial effect. Figure 5 shows the logarithmic relationship between the viable bacterial count and the WCA and that between the control and the four types of FPB-treated surfaces. The vertical axis shows the viable bacterial count in the washout solution obtained after the test. FPB-1 and FPB-2 showed hydrophilic properties with good water wettability. As the body of a bacterial cell is hydrophilic, bacteria adhere to hydrophilic surfaces (An and Friedman 1998; Raut et al. 2010). Therefore, it is considered that it has an antibacterial effect against bacteria adhering to the surface of FPB. On the other hand, the bactericidal effects of nanopillar structures were stronger on hydrophobic surfaces (Nakade et al. 2018). Therefore, we consider that antibacterial factors in our study are different with the bactericidal effects of nanopillar structures.

**Fig. 6:**
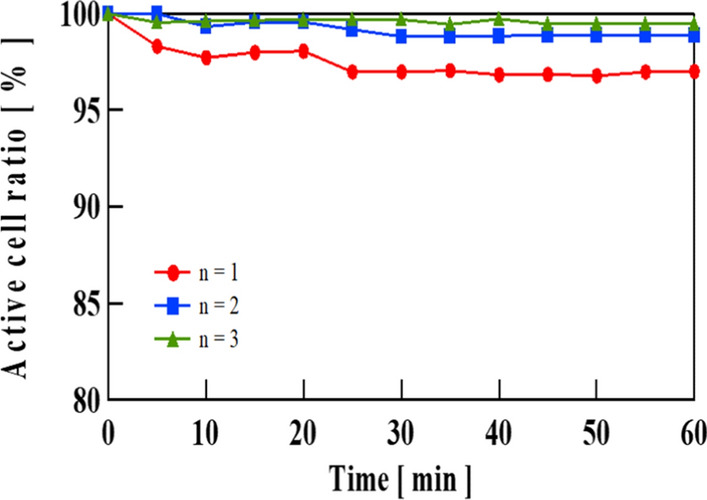
Variation in active cell ratio of FPB-2 with time, obtained from the results of live/dead assay test. Number of samples (N) was 3

**Fig. 7:**
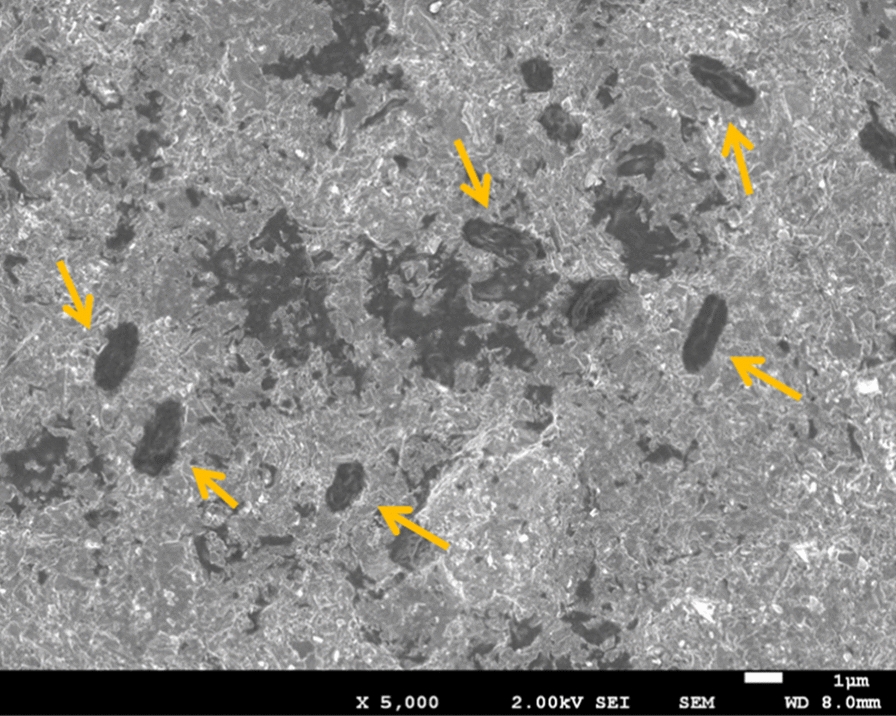
SEM image of the FPB-2 treated surface 60 min after the adhesion of *E. coli*. Arrows indicate *E. coli*

According to previous reports on the bactericidal effects of a nanopillar structure with an uneven pitch of approximately 200 nm, *E. coli* adhered to the convex part of the nanopillar structure. The interaction between *E. coli* and the nanostructure damaged the cell membrane. However, as the roughness pitch formed in this study was larger than the size of *E. coli*, it is unlikely that the aforementioned interaction occurred. To check whether the membrane damage occurred on the FPB treated samples, live/dead assay test was applied to FPB-2, for an example. The results are shown in Fig. 6. FPB-2 showed almost no decrease in the membrane damage rate up to 60 min after the attachment of *E. coli*. Furthermore, the scanning electron microscope images shown in Fig. 7 confirmed the intact morphology of *E. coli* on the surface of FPB-2. This further supported the idea that the antibacterial effect of the FPB-treated surfaces was not due to membrane damage.

Next, based on the test results for *S. aureus*, it achieved an antibacterial activity score of 2.0, even for a test duration of 6 h. We consider that the lower antibacterial effect of FPB-1 compared to FPB-2 was due to the roughness pitch size of the surface profile. The diameter of *S. aureus* is approximately about 1 µm. The roughness pitch of FPB-1 ranged from 0.4 to 1 µm, and its average roughness pitch was smaller than the bacterial cell diameter. In contrast, the roughness pitch of FPB-2 ranged from 1.7 to 7.3 μm, which was bigger than the bacterial cell diameter. According to the results of the antibacterial property test against *E. coli*, the antibacterial effect was likely enhanced by the relationship between roughness pitch and body length of the bacteria. Bagherifard et al. reported the suppression of the growth of *S. aureus* on irregular fine rough surfaces formed by shot peening (Bagherifard et al. 2015). The shot peening is a surface modification treatment method that uses particle size lager than FPB. However, the effect was lower than that observed in our study. This could be because of the difference in the roughness pitch. As a larger particle size (120 or 580 µm) compared to that in our study (several micrometers or less) was used previously, the roughness pitch formed by FPB was small. The roughness pitch of FPB-1 and FPB-2 correspond to the body length scale of a single bacteria. Based on this fact and results of the live/dead assay test, the mechanism of the antibacterial effect of FPB-1 and FPB-2 is thought to be that the bacteria are trapped in the concavity, which physically inhibits their movement, resulting in the inhibition of biofilm formation (Wood 2009) and cell division (Additional file 4: Figure S4). Although FPB-3 and FPB-4 have roughness pitch of 20–200 um, which do not correspond to the length scale of bacteria, a certain antibacterial effect was observed. In FPB treatment, an irregular roughness pitch is formed. This suggests that a certain percentage of the roughness pitch is consistent with the body length scale of a single bacteria, which may contribute to the antibacterial effect. In addition, multiple bacteria are thought to be trapped in concavity with a roughness pitch larger than the body length scale. In this case, too, the physical movement of the bacteria is inhibited to a certain extent by the concavity and convexity, and the bacteria is thought to exhibit growth inhibition. However, the details of this mechanism remain unclear. In the future, we expect to further investigate and clarify the mechanism of the antibacterial effect.

We evaluated that a stainless-steel substrate treated with FPB expressed antibacterial effects against *S. aureus* and *E. coli.* This result indicates that antibacterial effects were observed when the average pitch of convex pattern was lower than or equivalent to the size of bacteria. In addition, these substrates showed hydrophilicity and their WCAs were lower than 90°. From the live/dead assay and SEM observation, the membrane of *E. coli* was not damaged on the FPB-treated surface. The results suggested that the mechanism of the antibacterial effects of FPB-treated surfaces was different from the nanopillar-based antibacterial mechanism. Nanopillar surfaces have been shown to induce membrane damage in bacteria, whereas with FPB-treated surfaces, a strong antibacterial effect was obtained by forming a concavity that matched the size of a bacterium. The detailed mechanism of antibacterial effects remains unclear. FPB-treated surfaces are expected to have antibacterial effects on other bacteria which were not observed in this study. In the future, we will examine the antibacterial effects of FPB-treated surfaces on other types of bacteria and the antiviral effects of FPB-treated surfaces.

## Supplementary Information

**Additional file 1: Fig. S1**. Schematic image of FPB.

**Additional file 2: Fig. S2**. Schematic diagram of the plastic deformation on the FPB treated surface due to a single bombardment of fine particle on the substrate. The distance between the convexity to convexity is defined as the roughness pitch.

**Additional file 3: Fig. S3**. Relationship between the contact angle and roughness pitch of FPB-treated surfaces.

**Additional file 4: Fig. S4.** SEM image of the FPB-2 treated surface. S4(a) shows the SEM image of the FPB surface at a higher magnification than the Fig. 7. S4(b) shows the composite image of the trapped bacteria and the SEM image of Fig. S4(a).

**Additional file 5: Table S1**. Conditions for fine particle bombarding.

## Data Availability

The datasets during and/or analysed during the current study available from the corresponding author on reasonable request.
