# Supplementary figures and images for: Antibacterial effect on microscale rough surface formed by fine particle bombarding
Source: AMB Express. 2022 Jan 31;12:9. doi: 10.1186/s13568-022-01351-8 (PMC8804057; doi:10.1186/s13568-022-01351-8)

## Slide 1
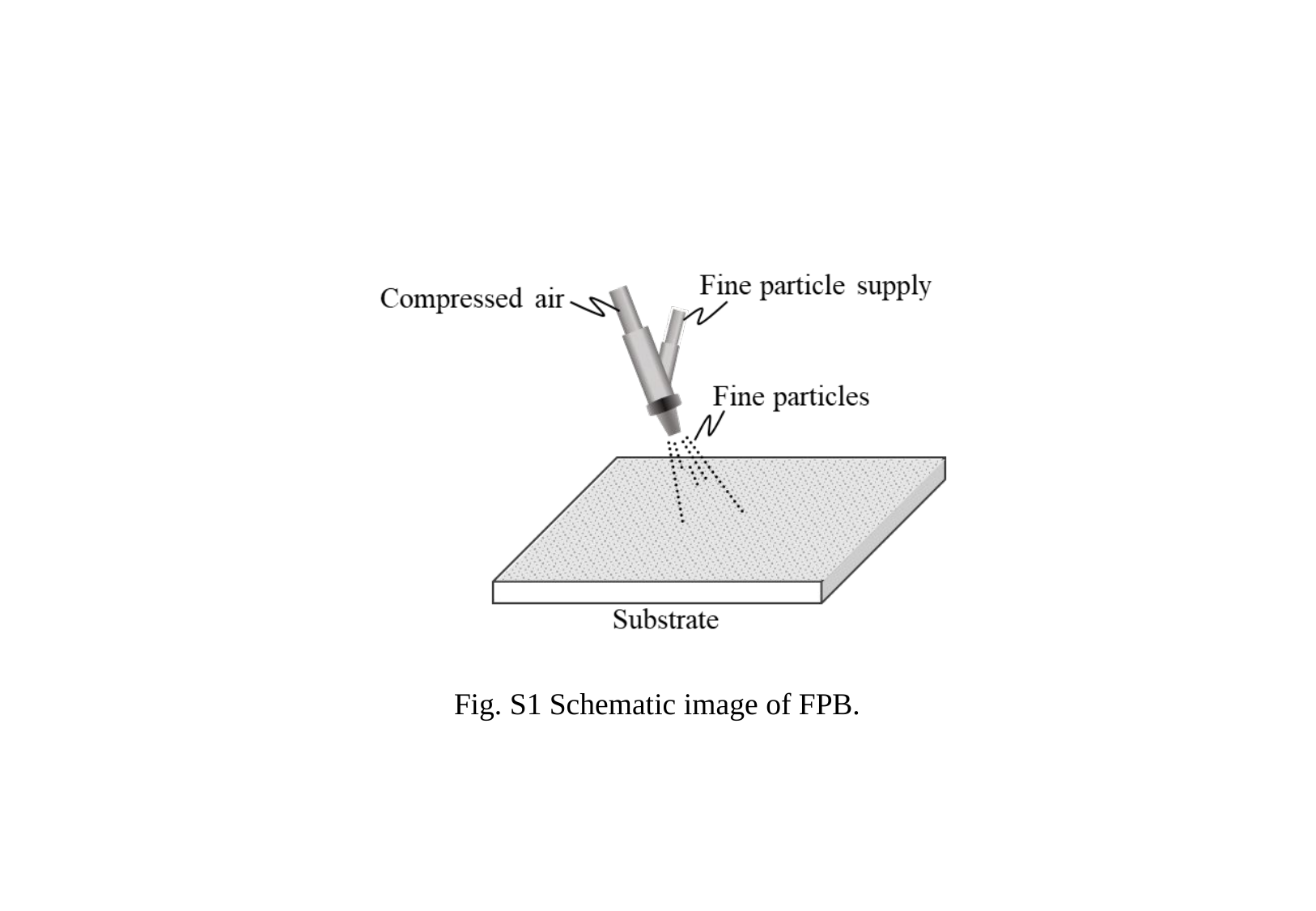

Fig. S1 Schematic image of FPB.

Supplement: Supplementary file 1 — Additional file 1: Fig. S1. Schematic image of FPB. [file 13568_2022_1351_MOESM1_ESM.pptx]
