# Supplementary material for: Antibacterial effect on microscale rough surface formed by fine particle bombarding
Source: AMB Express. 2022 Jan 31;12:9. doi: 10.1186/s13568-022-01351-8 (PMC8804057; doi:10.1186/s13568-022-01351-8)
Supplement: Supplementary file 2 — Additional file 2: Fig. S2. Schematic diagram of the plastic deformation on the FPB treated surface due to a single bombardment of fine particle on the substrate. The distance between the convexity to convexity is defined as the roughness pitch. [file 13568_2022_1351_MOESM2_ESM.pptx]

## Slide 1
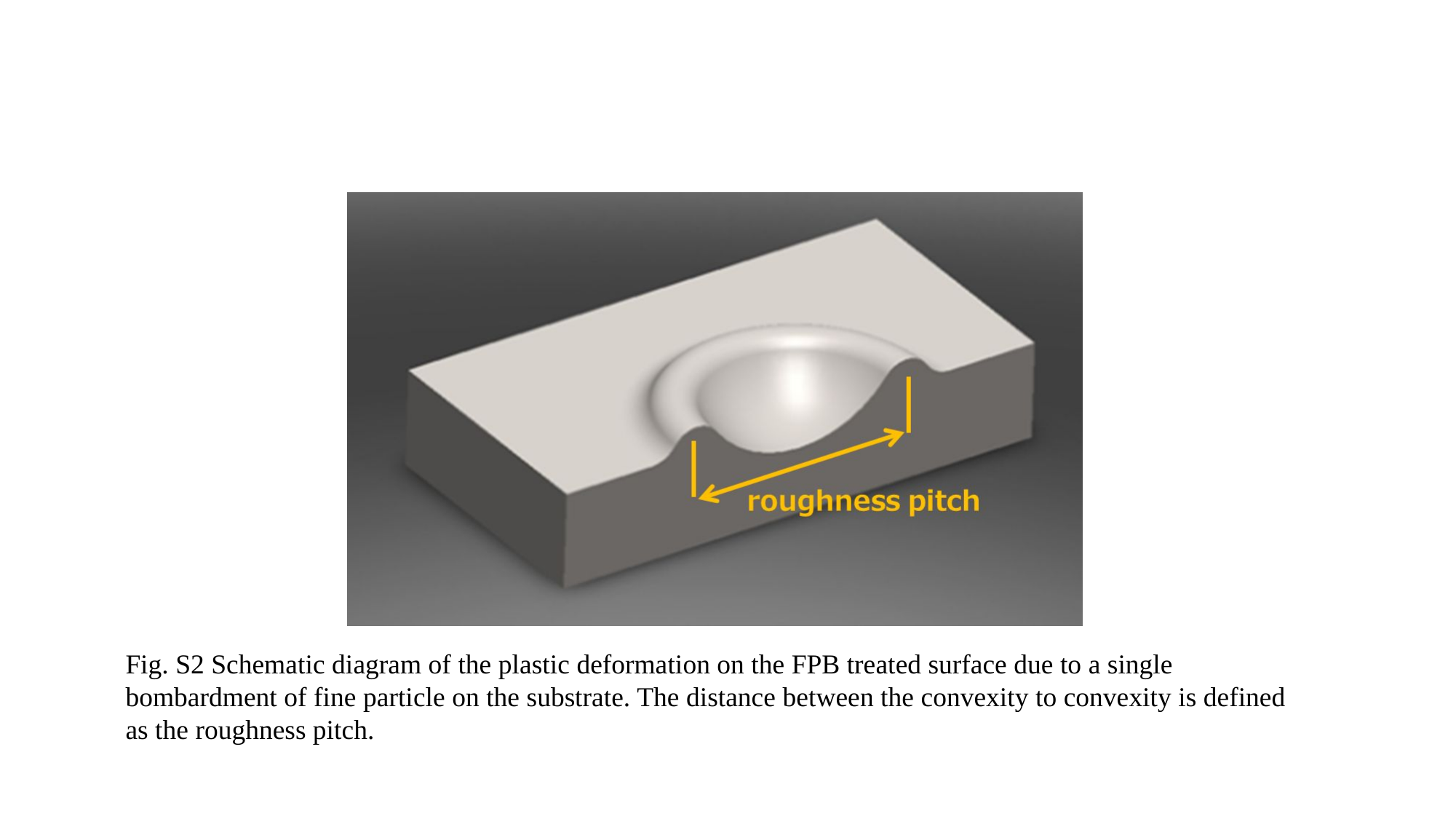

Fig. S2 Schematic diagram of the plastic deformation on the FPB treated surface due to a single bombardment of fine particle on the substrate. The distance between the convexity to convexity is defined as the roughness pitch.
