# Supplementary material for: Antibacterial effect on microscale rough surface formed by fine particle bombarding
Source: AMB Express. 2022 Jan 31;12:9. doi: 10.1186/s13568-022-01351-8 (PMC8804057; doi:10.1186/s13568-022-01351-8)
Supplement: Supplementary file 3 — Additional file 3: Fig. S3. Relationship between the contact angle and roughness pitch of FPB-treated surfaces. [file 13568_2022_1351_MOESM3_ESM.pptx]

## Slide 1
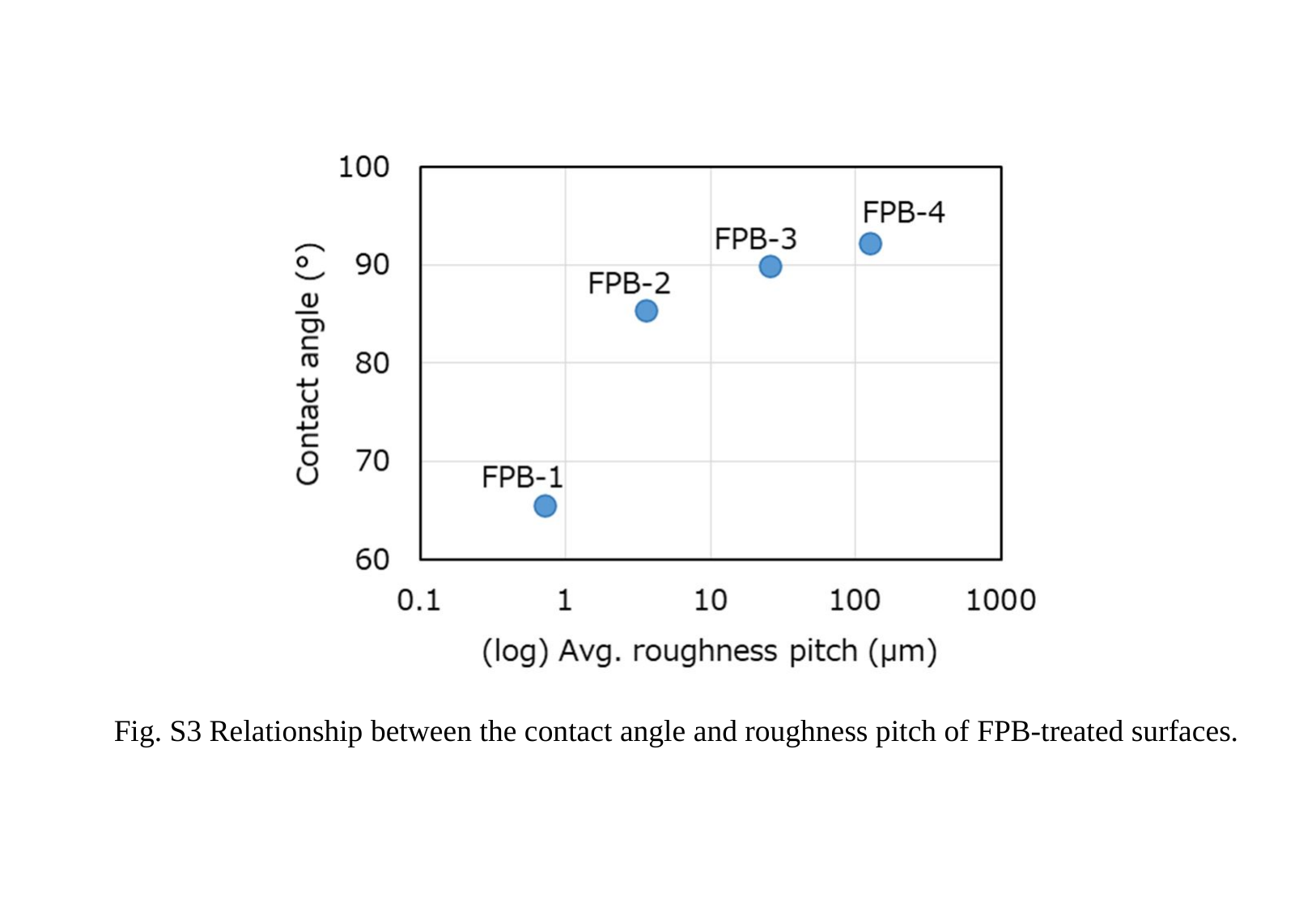

Fig. S3 Relationship between the contact angle and roughness pitch of FPB-treated surfaces.
