# Supplementary material for: Antibacterial effect on microscale rough surface formed by fine particle bombarding
Source: AMB Express. 2022 Jan 31;12:9. doi: 10.1186/s13568-022-01351-8 (PMC8804057; doi:10.1186/s13568-022-01351-8)
Supplement: Supplementary file 4 — Additional file 4: Fig. S4. SEM image of the FPB-2 treated surface. S4(a) shows the SEM image of the FPB surface at a higher magnification than the Fig. 7. S4(b) shows the composite image of the trapped bacteria and the SEM image of Fig. S4(a). [file 13568_2022_1351_MOESM4_ESM.pptx]

## Slide 1
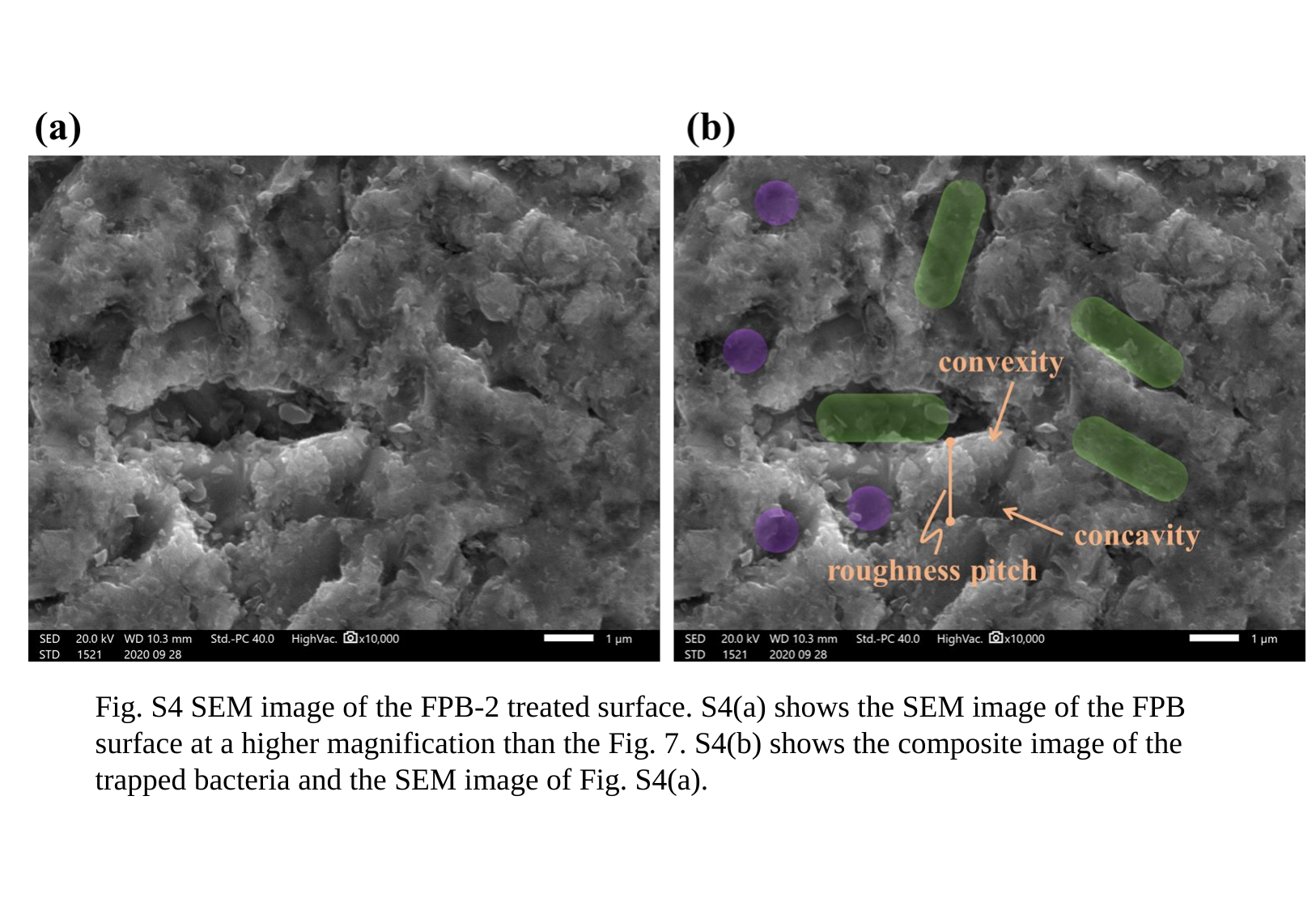

Fig. S4 SEM image of the FPB-2 treated surface. S4(a) shows the SEM image of the FPB surface at a higher magnification than the Fig. 7. S4(b) shows the composite image of the trapped bacteria and the SEM image of Fig. S4(a).
