# Supplementary material for: Antibacterial effect on microscale rough surface formed by fine particle bombarding
Source: AMB Express. 2022 Jan 31;12:9. doi: 10.1186/s13568-022-01351-8 (PMC8804057; doi:10.1186/s13568-022-01351-8)
Supplement: Supplementary file 5 — Additional file 5: Table S1. Conditions for fine particle bombarding. [file 13568_2022_1351_MOESM5_ESM.pptx]

## Slide 1
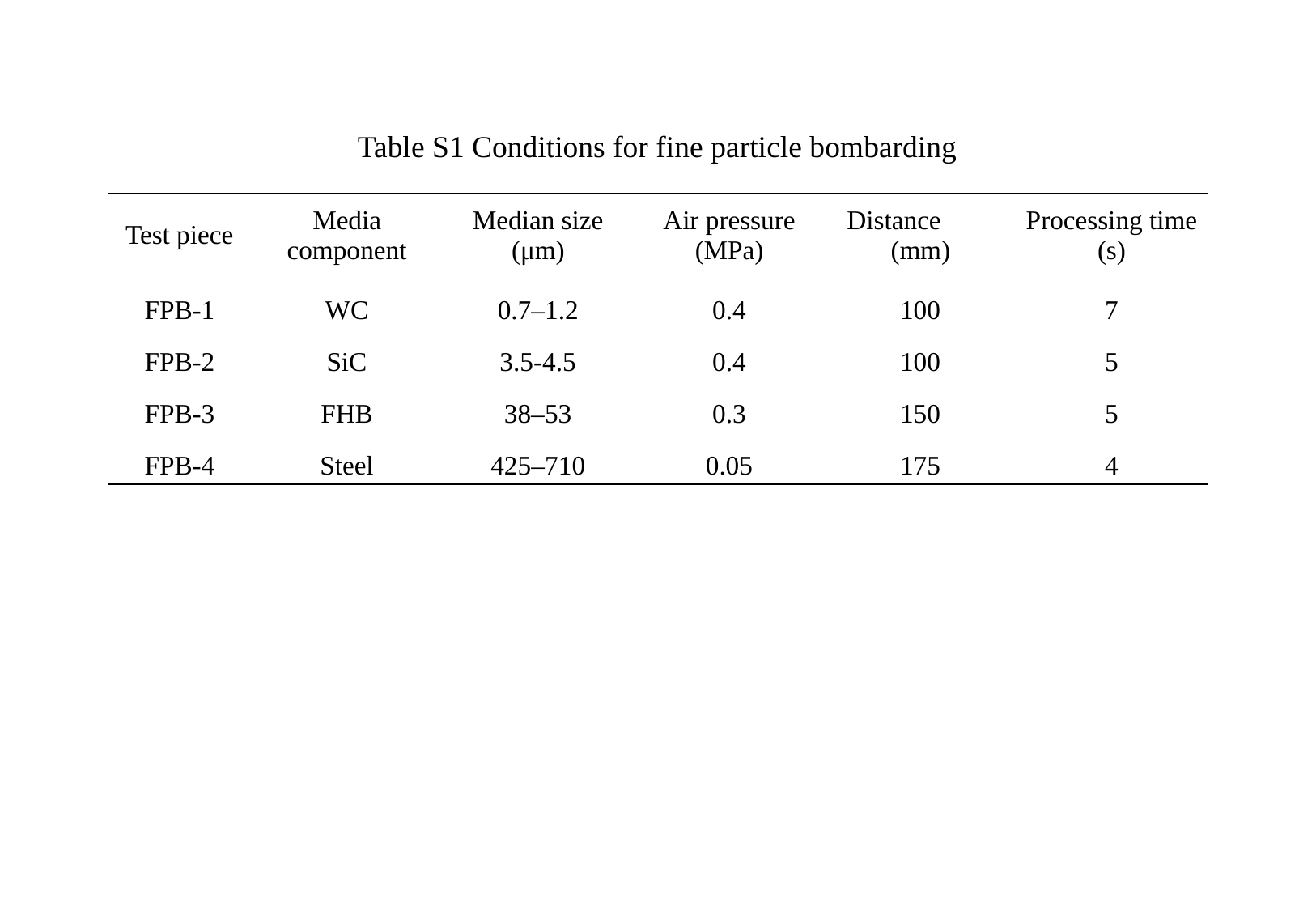

Table S1 Conditions for fine particle bombarding
| Test piece | Media component | Median size (μm) | Air pressure (MPa) | Distance 　 (mm) | Processing time (s) |
| --- | --- | --- | --- | --- | --- |
| FPB-1 | WC | 0.7–1.2 | 0.4 | 100 | 7 |
| FPB-2 | SiC | 3.5-4.5 | 0.4 | 100 | 5 |
| FPB-3 | FHB | 38–53 | 0.3 | 150 | 5 |
| FPB-4 | Steel | 425–710 | 0.05 | 175 | 4 |
